# Optimism and symptoms of anxiety and depression among Chinese women with breast cancer: the serial mediating effect of perceived social support and benefit finding

**DOI:** 10.1186/s12888-022-04261-y

**Published:** 2022-10-05

**Authors:** Qingqian Mo, Chen Tan, Xiang Wang, Tamini Soondrum, Jinqiang Zhang

**Affiliations:** 1 Department of Clinical Psychology, The Third Xiangya Hospital of Central South University, Changsha, Hunan 410013 People’s Republic of China; 2 Medical Psychological Center, The Second Xiangya Hospital of Central South University, Changsha, Hunan 410000 People’s Republic of China

**Keywords:** Optimism, Perceived social support, Benefit finding, Anxiety, Depression, Breast cancer, Serial mediation

## Abstract

**Objective:**

This research examines the direct and indirect relationships between optimism, perceived social support (PSS), benefit finding (BF), and anxiety and depressive symptoms among Chinese women with breast cancer (BC).

**Methods:**

We recruited 512 patients, aged averagely 47.46(*SD* = 8.51) years from two hospitals located in Hunan province, China. The variables were assessed using the Optimism–Pessimism Scale (OPS), the Multidimensional Scale of Perceived Social Support (MSPSS), the Benefit Finding Scale (BFS), and the Hospital Anxiety and Depression Scale (HADS). Path analyses were conducted by Amos version 24.0 for Windows to test the hypothesized serial mediation model.

**Results:**

Path analyses suggest a significant negative association between optimism and symptoms of anxiety and depression. The relationship was mediated by BF (*β* = -0.085, *SE* = 0.015, 95% CI [-0.126, -0.055]), and by BF together with PSS (*β* = -0.027, *SE* = 0.007, 95% CI [-0.047, -0.017]). The difference comparison between the two indirect effects was significant (*β* = 0.057, *SE* = 0.015, 95% CI [0.034,0.101]).

**Conclusions:**

Our findings suggest that PSS, and BF are important mediators through which optimism may buffer symptoms of anxiety and depression among Chinese BC patients. Clinicians and healthcare practitioners should be aware of the importance of patients’ emotional health and endeavor to offer emotional support, facilitate their capacity to improve their quality of life.

## Introduction

Breast cancer (BC) is considered the most commonly diagnosed cancer in women worldwide [1, 2]. According to the China National Cancer Center, China accounts for 12.2% and 9.6% of new breast cancer cases and deaths, respectively. The prevalence rate in China is exponentially contributing to the global prevalence [3]. With the development of medical technology, the survival rate of breast cancer worldwide has improved significantly [4]. However, the adverse consequences of cancer and its treatments continue to plague patients, which interferes with patients' state and quality of life [5].

Notably, a growing body of literature recognizes the importance of improving the quality of life alongside the disease, particularly addressing emotional distress, such as symptoms of depression and anxiety [6]. Recent evidence suggests that failure to identify and treat symptoms of anxiety and depression can increase the potential risk of poor quality of life, disease-related morbidity, and mortality [7]. From a resource-oriented belief, this may be because patients will face huge challenges in adapting resources. They were related to the social and interpersonal relations (i.e., family relations, social support, and work) and personal internal resources (i.e., dispositions, individual traits, cognitive mechanisms, and abilities) [8]. It is important to identify individual differences and psychological resources related to positive physical and mental health results, which can promote interventions which improve the health and daily function of cancer patients.

Optimism is a key personality resource that has proven to be a positive factor for individuals coping with troubles in life [9]. Dispositional optimism defined by Scheier and Carver is a cognitive-affective personality construction. It reflects individuals’ universal and stable expectations that something good will happen to them [10]. Besides, it functions as a personal psychological resource that can bring health benefits [11, 12]. Extensive research has shown that a high level of dispositional optimism is a protective factor for cancer survivors' quality of life, sexual impairment, well-being, fatigue, symptoms of anxiety and depression [13–16]. While low optimism has been confirmed to be a significant predictor of psychological distress [17]. Many studies suggest a significant negative association between optimism and negative emotions [18, 19].

Optimistic patients show greater satisfaction with support after surgery [20]. Perceived social support defined by Procidano and Heller, is the belief that support is accessible if there is a need [21]. Recent work has established that social support is a vital mediator working between optimism and regulating relationships. This could be because an optimistic personality can attract more people and allow individuals to establish more relationships, thereby augmenting social support [22, 23]. Besides, it has been evidenced that perceived social support mediates (partially or entirely) the association between optimism and better psychological adjustment among BC patients [24]. Perceived social support might be a significant mediator between optimism and symptoms of anxiety and depression among BC patients.

Benefit finding, another vital concept in adaptive resources, is a cognitive reappraisal coping strategy [25] or adaptation [26]. Benefit finding, defined by Affleck and Tennen, are positive life changes in stressful events like a cancer diagnosis [27]. It plays an important role in promoting BC patients to adapt to life when accompanied by diseases. Individuals can gain psychological benefits from their cancer experiences for better adaptive coping mechanisms. BC-related benefits such as sensing closer relationships with others and greater spirituality appear to become a common phenomenon [28]. One might report that confronting cancer prompted a realization of others’ supportiveness [29]. In addition, personality predictors of benefit finding include optimism and hope. Individuals who hold generalized expectancies for positive outcomes may seek opportunities to transform threatening situations into favorable circumstances through benefit finding [29]. Furthermore, some studies have yielded significant concurrent relations between perceived positive outcomes and better mood or quality of life [30]. In a longitudinal study, benefit finding predicts lower levels of distress after stressful event [31]. Based on a meta-analysis, the results implied that posttraumatic benefit findings predict positive markers of mental health (i.e., positive impact, self-esteem, life satisfaction, and less depression) [32].

Despite various studies have assessed the relationship among these variables, the underlying mechanisms are still not fully understood. According to these theories and previous findings, we hypothesized that optimism contributes to buffering symptoms of anxiety and depression. As individuals with high optimism could perceive more social support and find more benefits, it was further hypothesized to reduce symptoms of anxiety and depression. In other words, perceived social support and benefit findings mediate the association between optimism and symptoms of anxiety and depression.

Therefore, the current study aimed to examine the direct and indirect effects of optimism on symptoms of anxiety and depression through the roles of perceived social support and benefit findings. Considering the unique medical and personal conditions of the study participants, we added three variables that may influence anxiety, depressive symptoms to the model (a) age, (b) months since diagnosis, and (c) years of education.

**Fig. 1:**
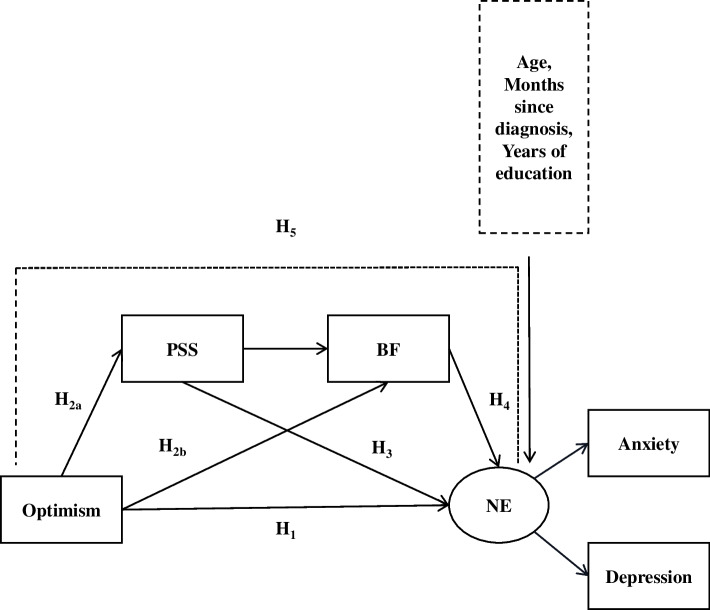
Hypothesized research model. Abbreviations: PSS, perceived social support; BF, benefit findings; NE, negative emotions (including symptoms of anxiety and depression)

The following hypotheses were formulated (Fig. 1):

- H_1_: Optimism negatively influences symptoms of anxiety and depression among BC patients.
- H_2_: Optimism positively influences perceived social support (H_2a_) and benefit findings (H_2b_).
- H_3_: Perceived social support negatively influences symptoms of anxiety and depression.
- H_4_: Benefit finding negatively influences symptoms of anxiety and depression.
- H_5_: Perceived social support and benefit finding are serial mediators between optimism and symptoms of anxiety and depression among BC patients.

## Methods

### Participants and procedure

We conducted a cross-sectional analytical study based on several self-reporting survey questionnaires. Between Oct-2014 to Nov-2019, we recruited women diagnosed with primary BC within the past week and undergoing curative treatment from Second Xiangya Hospital and Xiangya Hospital in Hunan province, China. Participants were eligible if they had histologically confirmed primary invasive carcinoma of the breast (stages I, II, and III) with no history of or current evidence of metastatic disease, 18 years and older, exhibited sufficient Chinese speaking and writing proficiency, without major psychiatric disorder or/and family history of mental disorders, without major somatic disease except for BC, without substance abuse history.

The study received ethical approval through the Ethics Committee of the Second Xiangya Hospital, Central South University, Hunan, China. Firstly, we orally informed the eligible women of the study’s purpose; those who agreed to participate then provided written informed consent. On enrollment, participants admitted to the hospital were administered the Optimism–Pessimism Scale (OPS) and the Multidimensional Scale of Perceived Social Support Scale (MSPSS) by trained graduate students majoring in psychology. They also completed a form for demographic and clinical information. When they were discharged from the hospital, participants completed the assessment of the 17-item Benefit Finding Scale (BFS) and Hospital Anxiety and Depression Scale (HADS).

Five hundred twenty-seven eligible Chinese women in total volunteered to participate in the study, and finally, 512 completed and returned all assessments, with a valid response rate of 97.2%.

### Measures

#### Demographic and clinical characteristics

These included age, years of schooling, permanent place of residence, occupational status, marital status, and household income. We also collected information through self-report on menstrual and menopausal status, current smoking, and alcohol use. Clinical characteristics included the BC stage, the surgery type, and the time since diagnosis.

#### Optimism–Pessimism Scale (OPS)

The Optimism–Pessimism Scale (OPS) is an 11-item self-report measure assessing optimism and pessimism [33]. The optimism subscale contains five items (item 2, 5, 6, 8, and 10), and the pessimism subscale contains six items (item 1, 3, 4, 7, 9, and 11). Respondents are rated on a five-point Likert-type scale (1 = strongly agree to 5 = strongly disagree). The total score on the scale is obtained by adding the reverse score of the optimism subscale and the original score of the pessimism subscale. The higher the total score of the scale, the more optimistic tendency, and the lower the total score, the more pessimistic tendency. Jie Xia validated the Chinese version of the Optimism and Pessimism Scale in a sample of 730 adult Chinese individuals and the total scale and optimism and pessimism factors demonstrated satisfactory reliability and validity [34]. In the present study, the Cronbach alpha coefficients of the total OPS scale and the optimistic, pessimistic subscales were 0.94, 0.91,0.89, respectively.

#### Multidimensional Scale of Perceived Social Support (MSPSS)

The Multidimensional Scale of Perceived Social Support (MSPSS) is a self-reported measure of twelve items used to rate perceived social support, consisting of three subscales that assess perceived social support from family (item 3, 4, 8, 11), friends (item 6, 7, 9, 12), and significant other (item 1, 2, 5, 10) [35]. Respondents are scored on a seven-point Likert-type scale (1 = strongly disagree to 7 = very strongly agree). MSPSS demonstrated excellent internal consistency in a Hong Kong Chinese adolescent sample [36]. In the present study, the Cronbach alpha coefficients for the MSPSS total scale and family, friends, and significant other subscales were 0.95, 0.90, 0.96, and 0.88, respectively.

#### Benefit Finding Scale (BFS)

The Benefit Finding Scale (BFS) is a 17-item self-report measure assessing the extent to which participants reported finding benefits from the experience of cancer [37]. Participants were asked to mark each item with a five-point Likert-type scale (1 = not at all to 5 = extremely), the extent to which each item applied to them. Higher values show that the participant gained more benefits from the cancer experience [38]. The Chinese version of BES translated by Zhunzhun Liu and have been well validated [39]. The Chinese BFS demonstrated good patient acceptability and exhibited strong psychometric properties among Chinese patients with early-stage cancer [39, 40]. In the present study, the Cronbach alpha coefficient was 0.97.

#### Hospital Anxiety and Depression Scale (HADS)

The Hospital Anxiety and Depression Scale (HADS) is a 14-item self-report measure assessing the severity of anxiety and depression symptoms over the prior week [41]. Items marked D indicate depression symptoms, and items marked A indicates anxiety symptoms. Respondents are rated on a four-point Likert-type scale (0–3). HADS has been introduced in many countries and applied to different groups of people in the world, such as Greek [42] and Spanish [43] in patients, samples of different ages in Dutch [44] and cancer patients and their family caregivers in China [45]. And all of them have reported a good internal consistency. In the present study, the Cronbach alpha coefficients for the HAD total scale and anxiety, depression subscale were 0.94, 0.87, 0.90, respectively.

### Statistical analysis

We performed statistical analyses using Statistical Package for Social Sciences Version 22.0 and Amos version 24.0 for Windows (SPSS Inc., Chicago, IL, USA). We first performed the Kolmogorov–Smirnov test for normality of the main study variables and found that they were all non-normally distributed (*p* < 0.05). Therefore, nonparametric Kruskal–Wallis analysis of variance test was conducted to compare benefit findings, anxiety, and depression for each categorical demographic and clinical variable for the descriptive analysis of sociodemographic and clinical characteristics of the participants. We included demographic and/or clinical variables which show significant group differences in the main study variables in the path analyses. Relationships between the primary study variables were analyzed through the analysis of correlation.

The proposed hypothesized research model presented in Fig. 1 was examined via path analysis using the structural equation modeling software Amos version 24.0 (IBM Corporation) [46]. Model fit was tested for optimism, perceived social support, benefit finding, negative emotions (including symptoms of anxiety and depression) separately. The PSS average score, OPS average score, BFS average score, age, and months since diagnoses represented their respective observed variable in the proposed model. Maximum likelihood estimation was used to assess model fit. Four estimates of model fit were used, based on suggested recommendations in the literature: (a) chi-square (χ^2^), (b) root mean square error of approximation (RMSEA), (c) normed fit index (NFI), and (d) comparative fit index (CFI). RMSEA index values at or above 1.0 indicate a poorly fitting model, values between 0.05 and 0.90 indicate an average model fit, and values below 0.05 indicate a superior model fit. CFI and NFI index values greater than 0.90 indicate a good model fit.

Bootstrap can be used for testing non-normal data. We conducted a bootstrap procedure with 5000 samples and a bias-corrected 95% confidence interval to examine indirect effects. An absence of zero in the 95% CI suggests a significant indirect effect.

## Results

### Sample characteristics

The 512 BC patients who completed all questionnaires were included in the analyses. The age of 512 participants ranged from 26 to 70 years (*M* = 47.46, *SD* = 8.51). On average, participants received 10.19 (*SD* = 3.52) years of education, completed BFS and HADS 1.34 months (*SD* = 5.48) since diagnosis. About half of the participants lived in urban (50.2%) and rural (49.8%) areas for a long time. Most (93.9%) were married, one woman was unmarried, 5.9% were widowed or divorced (Table 1).

**Table 1:** Summary of participant demographic and clinical characteristics

	Mean (*SD*)	Range	*N* (%)
Demographic information			
Age (years)	47.46 (8.51)	26–70	
< 50			348 (68.0)
≥ 50			164 (32.0)
Years of education	10.19 (3.52)	0–19	
Long-term place of residence			
Urban			257 (50.2)
Rural			255 (49.8)
Marital status			
Unmarried			1 (0.2)
Married			481 (93.9)
Widowed or divorced			30 (5.9)
Sex life			
Yes			392 (76.6)
No			120 (23.4)
Family monthly income			
Below 1000			28 (5.5)
1001–3000			173 (33.8)
3001–5000			150 (29.3)
5000–10,000			116 (22.7)
Above 10,000			45 (8.8)
Clinical information			
Months since diagnosis	1.34 (5.48)	0–82.77	
Stage			
Preoperative			163 (31.8)
Postoperative			327 (63.9)
Benign			22 (4.3)

### Descriptive analyses of main research variables

Table 2 presents the means, standard deviations, minimum, maximum, possible range and reliability measures of the primary research variables. Results from the Kruskal–Wallis analysis of variance tests indicated significant group differences in the demographic variable of marital status (married vs. widowed or divorced) in the MSPSS (*p* = 0.016) and Anxiety subscale (*p* = 0.005); long-term place of residence (urban vs. rural) in the OPS (*p* = 0.001), MSPSS (*p* < 0.001), BFS (p < 0.001), Anxiety subscale (*p* = 0.033) and Depression subscale (*p* < 0.001); age (below 50 years old vs. 50 years or older) in the MSPSS (*p* = 0.004), BFS (*p* = 0.032), Anxiety subscale (*p* = 0.006) and Depression subscale (*p* < 0.001). Additionally, there were significant group differences in categorical variable of Stage (preoperative vs. Postoperative vs. benign) in the OPS (*p* < 0.001), BFS (*p* = 0.035), Anxiety subscale (*p* < 0.001) and Depression subscale (*p* = 0.004).

**Table 2:** Descriptive statistics of main research variables (N = 512)

	Mean (*SD*)	Min	Max	Possible range	Cronbach alpha
OPS	39.05 (9.61)	19	55	11–55	0.94
MSPSS	64.95 (11.25)	21	84	7–84	0.95
BFS	32.11 (13.52)	17	79	17–85	0.97
HADS					
Anxiety	6.23 (3.91)	0	17	0–21	0.87
Depression	5.61 (4.21)	0	16	0–21	0.90

*Abbreviations*: *OPS* Optimism–Pessimism Scale, *MSPSS* Multidimensional Scale of Perceived Social Support, *BFS* Benefit Finding Scale, *HADS* Hospital Anxiety and Depression Scale

### Correlational analyses

Correlation sizes of main study variables were moderate, ranging from-0.235 to-0.484 (*p* < 0.01). The smallest correlations were observed between the MSPSS and the Anxiety subscale (*r* =-0.235), whereas the largest correlations were observed between the OPS and the Anxiety subscale and Depression subscale (*r* = -0.479 and -0.484, respectively) (Table 3).

**Table 3:** Correlations among the mean score of OPS, MSPSS, BFS, Anxiety subscale and Depression subscale (*N* = 512)

	1	2	3	4	5
1 OPS	–	0.346	0.438	-0.479	-0.484
2 MSPSS		–	0.425	-0.235	-0.318
3 BFS			–	-0.403	-0.417
4 Anxiety				–	0.871
5 Depression					–

All correlations significant at *p* < 0.001

### Path analyses

**Fig. 2:**
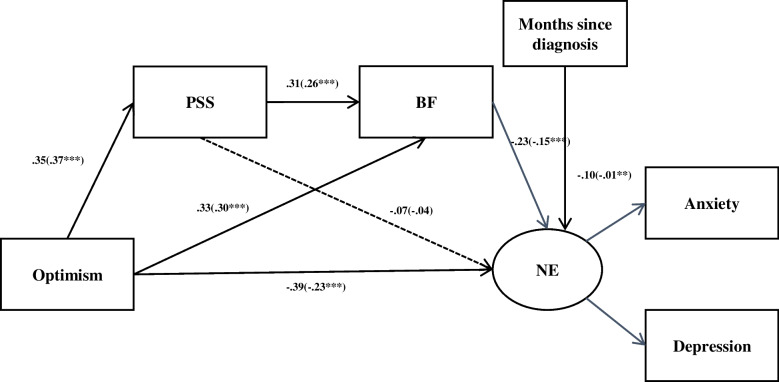
The final research model. *Note*: Structural equation model for the association between Optimism, Perceived social support (PSS), Benefit finding (BF), and Negative emotion (NE). Rectangles represent manifest variables(average score of the scale), Circles represent latent variables. Solid and dotted lines signify statistically significant and nonsignificant standardized path coefficients, respectively (**p ≤ 0.01; ***p ≤ 0.001)

In the path analysis NE (negative emotions) was used as the indicator of the observed variable of symptoms of Anxiety and Depressive. Results indicated that all pathways in both models were significant, with several exceptions: (a) Age to NE (*β* = -0.042, *p* = 0.275), (b)Years of education to NE (*β* = -0.031, *p* = 0.416), (c) PSS to NE(*β* = -0.07, *p* = 0.094) (Fig. 2).

The proposed model suggested a good fit to the data. Estimates of model fit were as follows: (a) *χ*^2^/*df* = 3.118 (*χ*^2^ = 21.829; *df* = 7), (b) RMSEA = 0.064, (c) NFI = 0.986, (d) CFI = 0.987. The model explained 12% of the variance in PSS, 27.9% of the variance in BF and 33% of variance in NE.

### Serial mediating analyses

The results of the bootstraps showed that Optimism have a significant total effect on NE (*β* = -0.514, *SE* = 0.035, 95%CI [-0.58, -0.443]) and a significant direct effect on NE (*β* = -0.402, *SE* = 0.043, 95%CI [-0.485, -0.318]) (Table 4). Moreover, the results revealed that benefit finding mediated the association between Optimism and NE (*β* = -0.085, *SE* = 0.018, 95% CI [-0.126, -0.055]). And the serial mediating effects of PSS and BF between Optimism and NE were also supported (*β* = -0.027, *SE* = 0.007, 95% CI [-0.047, -0.017]) (Table 5). Then, the difference comparison between the two indirect effects was conducted to test whether they exerted equal impacts on the association between optimism and NE. The results indicated that the indirect effect of Optimism on NE through BF was significantly greater than the serial mediating effect (*β* = 0.057, *SE* = 0.015, 95%CI [-0.034, -0.101]) (Table 5).

**Table 4:** Standardized Direct, Indirect, and Total Effects

	Point Estimate	Product of Coefficients		Bootstrapping	
Optimism → NE				Bias-Corrected 95%CI	
	*β*	*SE*	*Z*	Lower	Upper
Total Effects	-0.514	0.035	-14.686	-0.58	-0.443
Direct Effects	-0.402	0.043	-9.349	-0.485	-0.318
Indirect Effects	-0.112	0.023	-4.870	-0.155	-0.07

*β* Standardized Beta, *SE* Standard Error, *CI* Confidence Intervals

**Table 5:** Standardized Indirect Effects and Diff comparison

	Point Estimate	Product of Coefficients		Bootstrapping	
				Bias-Corrected 95% CI	
	*β*	*SE*	*Z*	Lower	Upper
Optimism → PSS → BF → NE	-0.027	0.007	-3.857	-0.047	-0.017
Optimism → BF → NE	-0.085	0.018	-4.722	-0.126	-0.055
Difference comparison	0.057	0.015	3.800	0.034	0.101

*β* Standardized Beta, *SE* Standard Error, *CI* Confidence Intervals

## Discussion

Past studies have consistently shown a significant link between optimism and emotional distress, like anxiety and depression [25, 26]. However, little is known about the mechanism involved. In the field of both interpersonal and intrapersonal resources, we examined the relationship between optimism and negative emotions and further explored the possible avenues behind this association. The results showed that optimism impacted negative emotions directly and indirectly by way of perceived social support and benefit findings. As a mediation framework, these results have theoretical and practical significance. Clinicians in the field should actively pay attention to the symptoms of anxiety and depression of breast cancer patients, help patients learn to use available interpersonal resources and appropriate cognitive strategies, and encourage patients to develop more optimism towards a potential recovery, thus improving the patient's prognosis and quality of life.

There is a significant negative correlation between optimism and negative emotions, with the association being mediated independently and cumulatively through perceived social support and benefit findings. Although our research is the first to explore this relationship specifically among BC patients. The current findings were consistent with the established bodies of literature on these associations. First, as hypothesized, optimism negatively correlated with negative emotions. It is consistent with previous studies, which indicated that optimism has been shown to prevent emotional distress among women facing chronic stressors such as cancer diagnosis and treatment [47].

Second, optimism was positively correlated with perceived social support and benefit findings. In terms of social support, this may be because optimistic individuals could attract more people, let them build more relationships, and increase social support in a timely manner [22, 23]. The causal role of optimism in benefit findings has been demonstrated by Affleck et al. (1987) as optimism being an important pre-requisite capacity for individuals to find benefits [48]. The literature related to optimism shows that those optimistic about life are more likely to gain a sense of profit or gain, even if they are experiencing traumatic events [26].

In addition, we found a positive correlation between social support and benefit findings. An explanation for this might be that social and emotional support from supportive friends and family members can provide an important emotional cushion, empowering people to obtain more meaningful and beneficial positive responses, and redefine the situation as less threatening [49].

Contrary to expectations, social support could not negatively influence negative emotions, nor can social support mediate the association between optimism and negative emotions. The findings are consistent with those reported by Shelby et al. (2008), which indicate that social support might be an important protective resource for low-optimistic women. While among high-optimistic women, increasing social support does not bring added benefits [50]. In other words, social support could play a moderating role rather than a mediating role in the association between optimism and negative emotions. Women with high levels of social support could adapt better even if they are less optimistic, which implies that social support can compensate for low levels of optimism.

In general, our results suggested that optimism could influence perceived social support, benefit findings, and negative emotions. Moreover, perceived social support could influence benefit findings, and benefit findings could influence negative emotions. These findings indicate that optimism seems to work via interpersonal and cognitive resources to influence negative emotions. Ultimately, as we expected, a serial-mediation model with social support as a prerequisite for benefit findings was found. The finding that social support is a precursor are as follows: Previous research has shown that perceiving more social support allows individuals to seek out and find their beneficial benefits, and then reduce negative emotions. For instance, in a longitudinal study of benefit findings among cancer patients, those with more social support reported greater benefit findings [51].

### Clinical implications

Our research has thrown some new light on the understanding of negative emotions for BC patients in adapting to diseases. We use a serial mediation framework model to explore the relationship between optimism, perceived social support, benefit findings, and symptoms of anxiety and depression. The findings can be put into clinical practice to promote quality of life and facilitate treatment interventions for BC patients. On the one hand, our findings suggest a reduced capacity for low-optimistic individuals to perceive social support and identify benefit findings to and from cancer suffering, which leads to more emotional distress. Then we can take measures to identify and support this risky group of patients. On the other hand, optimism is often seen as a core, stable personality trait that is unlikely to change easily. If we want to take effective interventions, we can train and change patients' cognition and improve their ability to benefit from findings. For instance, Antoni et al. (2001) found that cognitive-behavioral stress management intervention (CBSM) could help women who were initially assessed as low-optimistic to find more benefits from cancer adversity [52]. In addition, it’s important to strengthen the education of the patient's family for them to provide more social support to the patient and help them better adapt to change. Finally, the possible differential effects of different treatment stages on anxiety and depressive symptoms require further attention and verification.

Three points can summarize the strengths of this research: (1) Integrating previous researches that only explored the relationship between optimism, perceived social support, benefit findings and symptoms of anxiety and depression, we simultaneously considered optimism (intrapersonal resource), social support (interpersonal resource), benefiting findings (cognitive resource), symptoms of anxiety and depression (negative emotions) to provide a comprehensive picture of the adaptive resource—emotional distress linkage. The serial mediation model provides us with new insights, enabling us to understand how personal traits affect interpersonal environment, then affecting cognitive strategy, and finally affect mental health. (2) We clarified the exact role of specific psychological resources, which is necessary when considering possible ways of targeted intervention (3). Given the close association between optimism and emotional distress after diagnosis, these findings also provide a viable way and explanation for the robust link between optimism and emotional distress, both from perspectives of reserved adaptive resources.

### Study limitations

Meanwhile, some limitations need to be mentioned. First of all, adopting a cross-sectional design in this exploratory study failed to conclude the causal relationship between these variables. Future studies using longitudinal methods are needed to further certify the direction from social support to symptoms of anxiety and depression, and to better understand how optimism affects symptoms of anxiety and depression. Second, it is only based on self-reported data of BC patients, and the research results are prone to have mono-method bias. Finally, data from our sample were derived from the Chinese population only. This limits generalizability to women who are not geographically similar.

## Conclusions

In conclusion, the present study enriches the literature about the association between optimism and symptoms of anxiety and depression among Chinese BC patients and demonstrates the potential psychological mechanisms underlying this association. Our findings suggest that optimism may reduce symptoms of anxiety and depression among Chinese BC patients through sensing more social support and finding more benefits from cancer. Psycho-social interventions and supportive care should focus on these adaptive resources to improve the quality of life of this group.

## Data Availability

The data supporting the findings of this study are available from the corresponding author upon reasonable request.

## Abbreviations

BC: Breast cancer
BF: Benefit findings
BFS: Benefit Findings Scale
CFI: Comparative Fit Index
CI: Confidence Interval
HADS: Hospital Anxiety and Depression Scale
MSPSS: Multidimensional Scale of Perceived Social Support
NE: Negative Emotions
NFI: Normed Fit Index
OPS: Optimism–Pessimism Scale
PSS: Perceived Social Support
RMSEA: Root Mean Square Error of Approximation
SD: Standard Deviation
SPSS: Statistical Package for Social Sciences
